# CRISPR/Cas9‐mediated knockout of *Ms1* enables the rapid generation of male‐sterile hexaploid wheat lines for use in hybrid seed production

**DOI:** 10.1111/pbi.13106

**Published:** 2019-04-11

**Authors:** Anzu Okada, Taj Arndell, Nikolai Borisjuk, Niharika Sharma, Nathan S. Watson‐Haigh, Elise J. Tucker, Ute Baumann, Peter Langridge, Ryan Whitford

**Affiliations:** ^1^ School of Agriculture, Food & Wine The University of Adelaide Urrbrae South Australia Australia; ^2^ Present address: School of Life Science Huaiyin Normal University Huai'an China; ^3^ Present address: New South Wales Department of Primary Industries Research Excellence Orange New South Wales Australia; ^4^ Present address: Commonwealth Scientific and Industrial Research Organisation, Agriculture and Food Urrbrae South Australia Australia

**Keywords:** CRISPR/Cas9, genome editing, hybrid wheat, Ms1, *Triticum aestivum*, male sterility

## Abstract

The development and adoption of hybrid seed technology have led to dramatic increases in agricultural productivity. However, it has been a challenge to develop a commercially viable platform for the production of hybrid wheat (*Triticum aestivum*) seed due to wheat's strong inbreeding habit. Recently, a novel platform for commercial hybrid seed production was described. This hybridization platform utilizes nuclear male sterility to force outcrossing and has been applied to maize and rice. With the recent molecular identification of the wheat male fertility gene *Ms1*, it is now possible to extend the use of this novel hybridization platform to wheat. In this report, we used the CRISPR/Cas9 system to generate heritable, targeted mutations in *Ms1*. The introduction of biallelic frameshift mutations into *Ms1* resulted in complete male sterility in wheat cultivars Fielder and Gladius, and several of the selected male‐sterile lines were potentially non‐transgenic. Our study demonstrates the utility of the CRISPR/Cas9 system for the rapid generation of male sterility in commercial wheat cultivars. This represents an important step towards capturing heterosis to improve wheat yields, through the production and use of hybrid seed on an industrial scale.

## Introduction

Global demand for food crops is projected to double between 2005 and 2050 (Tilman *et al*., 2011). In order to meet future demand and limit the environmental impact associated with doing so, new breeding technologies must be developed to increase crop yields (Tester and Langridge, 2010). Capturing heterosis through hybrid breeding is one of few crop improvement technologies that offers rapid and significant yield gains across diverse production environments. Hybrid seed has long been widely used for the production of major cereal crops such as maize and rice, but it has been a challenge to develop a commercially viable hybridization platform for bread wheat (*Triticum aestivum*) due to wheat's strong inbreeding features, and the absence of a simple, inexpensive and reliable system for hybrid seed production. Heterotic yield gains of more than 10% and enhanced yield stability have been observed in experimental wheat hybrids (Longin *et al*., 2013; Mühleisen *et al*., 2014), underscoring the potential of this breeding method. Given that wheat provides approximately one‐fifth of dietary calories and protein for the human population (Shiferaw *et al*., 2013), it is clear that the development of a viable wheat hybridization platform could have a substantial positive impact on global food security.

Commercial hybrid seed production requires efficient cross‐pollination between genetically distinct parental inbred lines. In addition, self‐pollination of the female parent must be avoided. In wheat, this has been difficult to achieve on a large scale due to a lack of efficient and reliable methods for separating the sexes and forcing outcrossing (Whitford *et al*., 2013). Recently, a novel hybridization platform that utilizes nuclear male sterility was described for maize (Wu *et al*., 2016). A key component of the platform is seed production technology (SPT), a process that enables the propagation of non‐transgenic nuclear male‐sterile inbred lines for use as female parents. This hybridization platform has since been extended to rice, a development that was made possible by the identification and isolation of the rice male fertility gene *OsNP1* (Chang *et al*., 2016). Recently, we identified the wheat male fertility gene *Ms1* by map‐based cloning and demonstrated its function via complementation of the EMS‐derived mutation *ms1d* (Tucker *et al*., 2017). In our previous report, we also described how *Ms1* could be used to establish SPT in wheat, and we highlighted the potential of genome editing for rapidly introducing highly penetrant recessive *ms1* alleles into elite wheat lines.

The CRISPR (clustered regularly interspaced short palindromic repeats)/Cas9 (CRISPR‐associated protein 9) system is currently the most widely used genome editing technology, largely due to its simplicity and flexibility. The system has two components that together form a ribonucleoprotein complex: the Cas9 endonuclease and a small guide RNA (gRNA). The gRNA contains a 20 nucleotide guide sequence that is designed to target a specific site (protospacer) in the genome via Watson–Crick base pairing. The protospacer must be located immediately 5′ to a protospacer adjacent motif (PAM), whose canonical form is 5′‐NGG‐3′ (Jinek *et al*., 2012). Following target site recognition, Cas9 creates a DNA double‐strand break (DSB). Repair of the DSB through the error‐prone non‐homologous end‐joining (NHEJ) pathway often leaves a lesion in the form of a small insertion/deletion (indel) mutation. Such mutations can shift the open reading frame of a coding sequence or introduce a pre‐mature stop codon, resulting in gene knockout.

Here, we used the CRISPR/Cas9 system to generate *Ms1* knockout wheat lines that exhibit male sterility in the first generation. One of the recessive *ms1* alleles was highly penetrant and stably transmitted to the T_1_, T_2_ and T_3_ generations. Our results demonstrate the utility of the CRISPR/Cas9 system for the rapid generation of nuclear male sterility in hexaploid wheat. We anticipate that this approach will facilitate the development of a commercially viable wheat hybridization platform.

## Results

### gRNA and vector design

**Figure 1:**
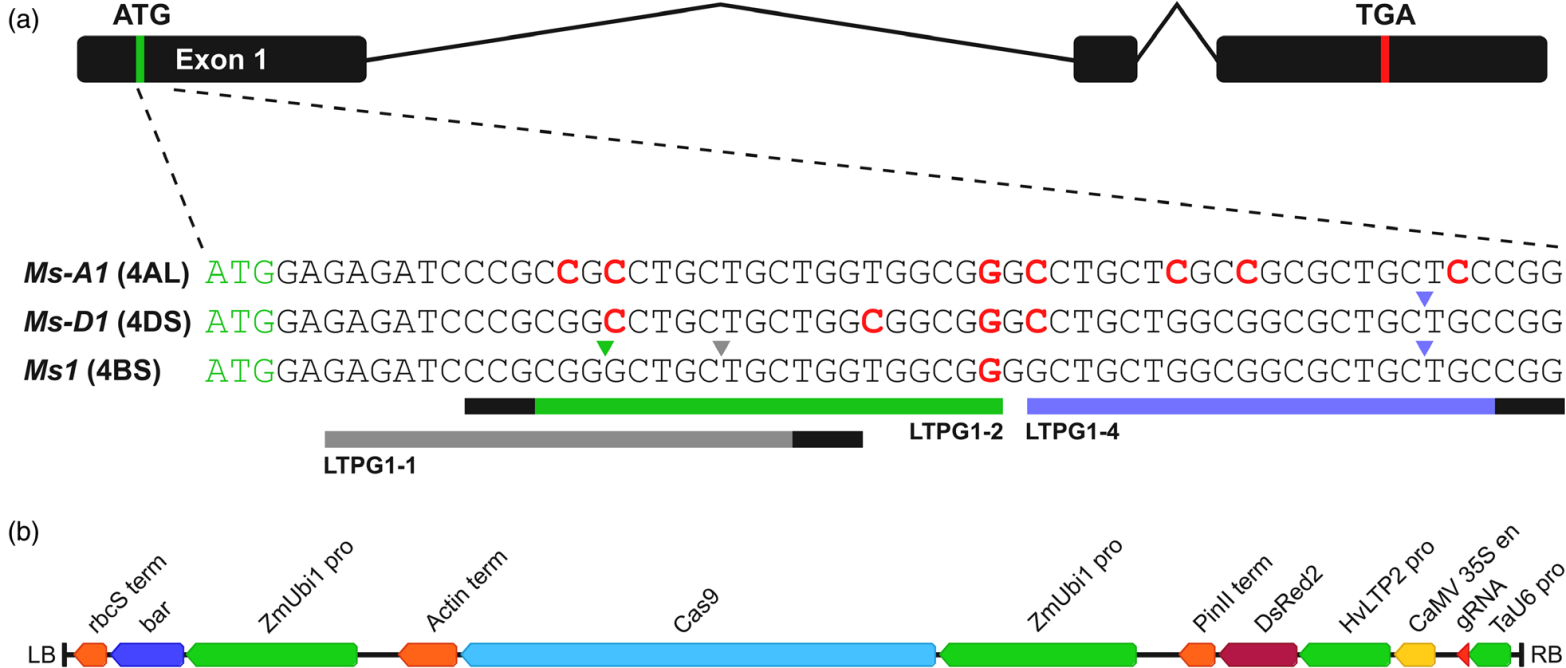
Target sites for gRNAs targeting *Ms1*, and the vector used for transformation. (a) The gene structure of *Ms1* and its homoeologs is shown. The detail underneath shows partial sequences for *Ms1* and its homoeologs, as well as the target sites for the three gRNAs (LTPG1‐1, LTPG1‐2 and LTPG1‐4) targeting exon 1 of *Ms1* on chromosome 4BS. Target sites are indicated by coloured bars (grey, green and purple). PAM sites (5′‐NGG‐3′) are indicated by black bars at the ends of the coloured bars. Downward‐pointing coloured arrow heads indicate the position of the canonical cut site, and the predicted specificity of the gRNA based on the number and distribution of gRNA mismatches (red text). (b) T‐DNA (12.8 kb) of the *Agrobacterium* binary vector used for transformation. LB, left border; RB, right border.

Using partial *Ms1* (chromosome 4BS), *Ms‐A1* (4AL homoeolog) and *Ms‐D1* (4DS homoeolog) sequences derived from *T. aestivum* cultivars Fielder and Gladius (NCBI GenBank accessions MK039721, MK039722, MK039723, MK039724, MK039725, MK039726), we designed three gRNAs (LTPG1‐1, LTPG1‐2 and LTPG1‐4) that target exon 1 of *Ms1* (Figure 1a). All three gRNAs targeted a predicted signal peptide. LTPG1‐1 was mismatched on chromosomes 4AL (positions +8 and +10 upstream of the PAM in the protospacer) and 4DS (position +8). LTPG1‐2 was mismatched on chromosomes 4BS (position +20), 4AL (positions +2, +4 and +20) and 4DS (positions +4, +15 and +20). LTPG1‐4 was mismatched on chromosomes 4AL (positions +2, +11, +14 and +20) and 4DS (position +20). Given that single mismatches at the PAM‐distal end of the guide sequence (i.e. position +20) are well‐tolerated unlike mismatches at the PAM‐proximal end (Hsu *et al*., 2013), we expected that LTPG1‐1 and LTPG1‐2 would specifically target *Ms1* on 4BS, while LTPG1‐4 would target both *Ms1* and *Ms‐D1*. A schematic of the *Agrobacterium* T‐DNA binary vector used for transformation is shown in Figure 1b.

### Identification of transgenic T_0_ plants with targeted mutations in *Ms1*

**Figure 2:**
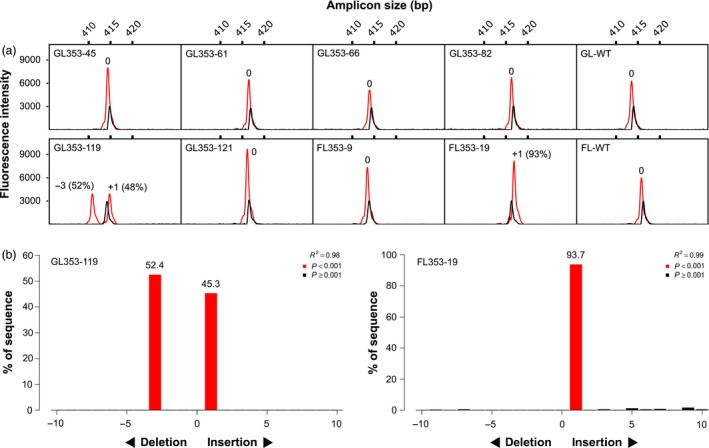
Screening of transgenic T_0_ wheat lines for targeted mutations. (a) Capillary separation of fluorescently labelled *Ms1*‐specific (chromosome 4BS) amplicons derived from eight representative lines carrying gRNA LTPG1‐2. A wild‐type control is also shown for each cultivar (GL‐WT, cv. Gladius; FL‐WT, cv. Fielder). Black peaks, wild‐type spike‐in (size reference); red peaks, transgenic or wild‐type (negative control) line. (b) TIDE analysis of Sanger sequence traces for the two putative biallelic mutants identified in (a).

**Figure 3:**
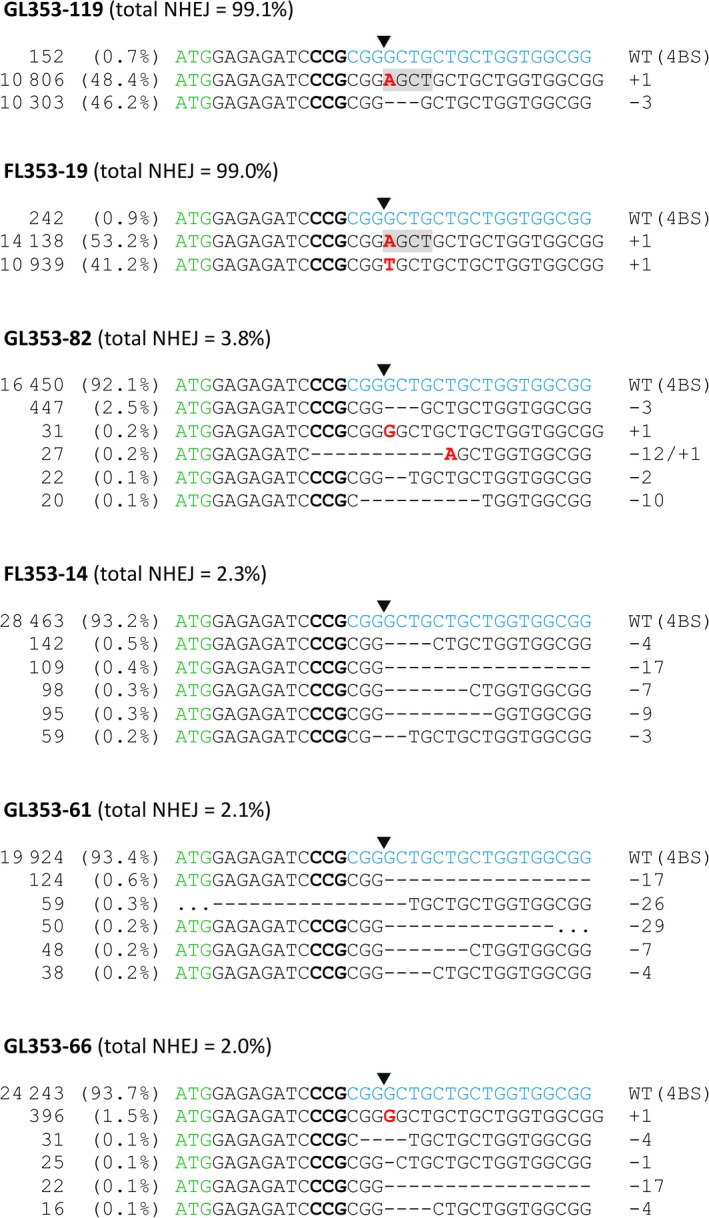
NGS reads from transgenic T_0_ mutant wheat lines carrying gRNA LTPG1‐2 targeting *Ms1* on chromosome 4BS. The number of reads is shown in the first column. The per cent of total reads is shown in the second column. Green text, start codon; bold text, PAM; blue text, target site. Downward‐pointing arrow heads indicate the position of the canonical cut site. *AluI* restriction sites are highlighted in grey.

We transformed 444 immature embryos of cv. Gladius and 352 immature embryos of cv. Fielder. We produced a total of 74 T_0_ transgenic lines (36 of cv. Gladius and 38 of cv. Fielder) carrying gRNA LTPG1‐1 (18 lines), LTPG1‐2 (40 lines) or LTPG1‐4 (16 lines). Transgene copy numbers ranged from 1 to 18 Table (S1). Two edited lines, both carrying LTPG1‐2, were identified by capillary separation of fluorescently labelled *Ms1*‐specific amplicons (Figure 2a). Line GL353‐119 (cv. Gladius, transgene copy number = 12) was a biallelic heterozygous mutant (+1/−3), and FL353‐19 (cv. Fielder, transgene copy number = 7) was a biallelic mutant (+1/+1). TIDE (Tracking of Indels by DEcomposition) analysis (Brinkman *et al*., 2014) of Sanger sequence traces (Data [Link], [Link], [Link], [Link], [Link], [Link], [Link], [Link], [Link], [Link], [Link], [Link], [Link], [Link]) confirmed the presence of targeted mutations in GL353‐119 (+1/−3) and FL353‐19 (+1/+1; Figure 2b). CRISPResso analysis (Pinello *et al*., 2016) of NGS reads (NCBI BioProject PRJNA495044) revealed that the targeted mutations in GL353‐119 and FL353‐19 were located precisely at the canonical cut site for LTPG1‐2 (Figure 3). In GL353‐119, the +1 insertion was an A nucleotide that created a new *AluI* restriction site, and the −3 deletion removed the Leu7 residue in the predicted signal peptide. In FL353‐19, the +1 insertions were A and T nucleotides. The CRISPResso analysis also identified several chimeric T_0_ lines in which <4% of genomic DNA was mutated at the target site (Figure 3). Of the three gRNAs, LTPG1‐2 induced targeted mutations in *Ms1* at the highest frequency, and it also had the highest WU‐CRISPR score (Wong *et al*., 2015) based on *in silico* predicted on‐target gRNA activity (Table 1). By contrast, LTPG1‐1 induced targeted mutations at very low frequencies, despite having the highest sgRNA Designer score (Doench *et al*., 2016; Table 1). None of the T_0_ lines contained off‐target mutations in *Ms‐A1* or *Ms‐D1* at a frequency above that observed in the wild‐type negative control (0.1%). GL353‐119 was partially male‐sterile (Figure S1), and FL353‐19 was fully male‐sterile, whereas all other T_0_ lines and the wild‐type negative controls were fully fertile.

**Table 1:** Summary of editing frequencies in T_0_ wheat lines carrying gRNAs targeting *Ms1* on 4BS

gRNA	Number of transgenic lines				*In silico* predicted gRNA activity	
	Total	>0.1% NHEJ	≥1.0% NHEJ	≥99% NHEJ	sgRNA Designer score*	WU‐CRISPR score†
LTPG1‐1	18	4 (22%)	0 (0%)	0 (0%)	0.4	<50
LTPG1‐2	40	28 (70%)	10 (25%)	2 (5%)	0.3	70
LTPG1‐4	16	0 (0%)	N/A	N/A	–	<50
None	N/A	0 (0%)	N/A	N/A	N/A	N/A

* Potential range of gRNA activity score = 0–1.

† Potential range of gRNA activity score = 0–100 (scores < 50 are not output); –, no score; N/A, not applicable. Editing frequencies (%NHEJ) are based on CRISPResso analysis of NGS reads. Wild‐type cv. Gladius was used as the negative control (bottom row).

### Inheritance of targeted mutations and male‐sterile phenotypes in the T_1_, T_2_ and T_3_ generations

To determine whether the targeted mutations were heritable, we tracked the inheritance of the +1 mutant allele in the progeny of the partially male‐sterile line GL353‐119 (+1/−3). As the +1 insertion of an A nucleotide created a new *AluI* restriction site, we were able to easily detect the mutant allele via *AluI* restriction enzyme assay.

**Figure 4:**
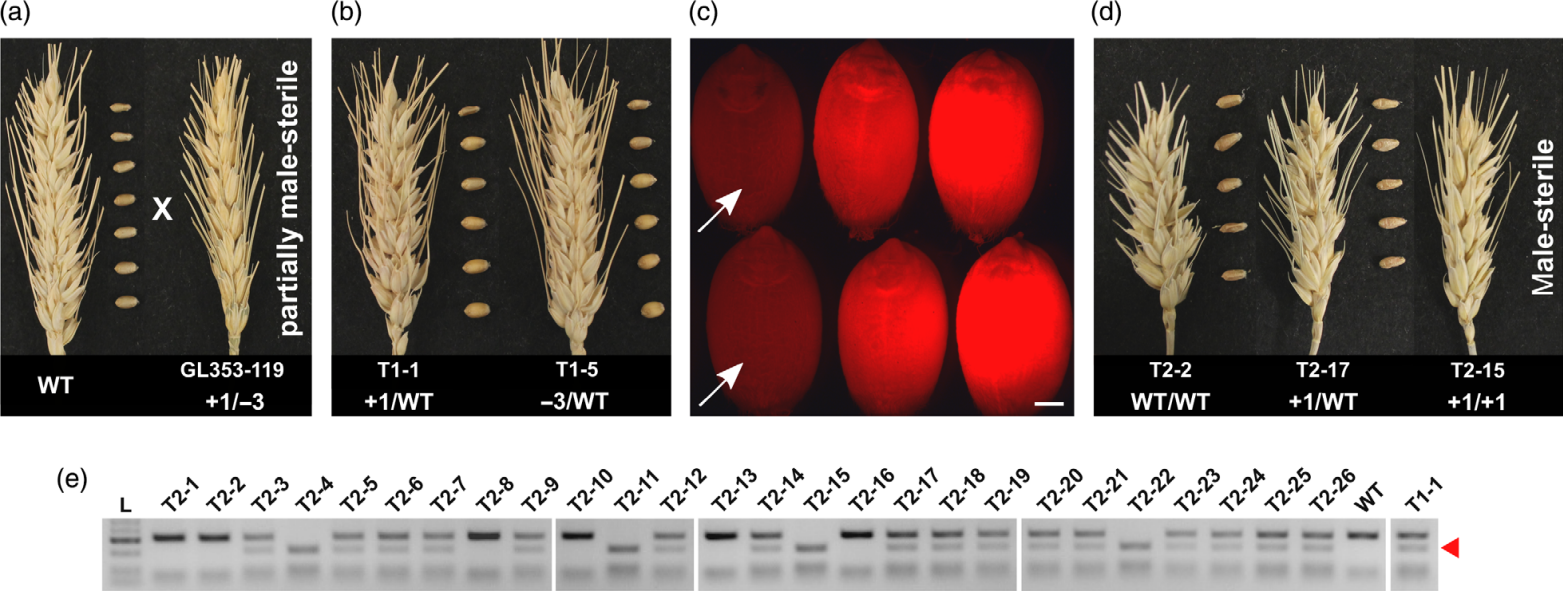
Inheritance and segregation of targeted mutations and male sterility generated with gRNA LTPG1‐2. (a) Crossing of the transgenic T_0_ mutant line GL353‐119 with wild‐type cv. Gladius. (b) Representative examples of T_1_ progeny derived from the cross shown in (a). The T_1_ progeny were selfed to produce T_2_ seeds. (c) Selection of DsRed‐negative (presumed non‐transgenic) T_2_ seeds produced by line T1‐1. Scale bar = 1 mm. (d) Representative examples of T_2_ progeny grown from the selected DsRed‐negative seeds. (e) Genotyping of the 26 DsRed/*Cas9*‐negative T_2_ progeny. The +1 mutant allele contains an *AluI* restriction site that is not present in the WT allele. Cleavage at the *AluI* restriction site results in a 210 bp band (red arrow head).

By crossing GL353‐119 (+1/−3) with wild‐type cv. Gladius (Figure 4a), we obtained T_1_ progeny (+1/WT or −3/WT), all of which were fully fertile (Figure 4b). Line T1‐1 (+1/WT) was selfed to produce 94 T_2_ seeds that were either DsRed‐positive (63 seeds) or DsRed‐negative (31 seeds), based on fluorescence microscopy (Figure 4c). The Cas9 transgene was detected by PCR in 59 of the 63 DsRed‐positive T_2_ plants (94%), while it was detected in only 5 of the 31 DsRed‐negative T_2_ plants (16%). The 26 DsRed/*Cas9*‐negative T_2_ plants were genotyped for the +1 allele and phenotyped for fertility. All of the WT/WT and +1/WT plants were fully fertile, whereas all of the +1/+1 plants were male‐sterile and produced no seed (Figure 4d and Table 2). The recessive +1 allele was inherited in a Mendelian fashion (Figure 4e and Table 2). Thus, we obtained four male‐sterile T_2_ plants that were apparently non‐transgenic (Table 2). Line T2‐14 (+1/WT) was randomly selected and selfed to produce T_3_ progeny. Fifty T_3_ plants were genotyped and phenotyped. All of the WT/WT and +1/WT plants were fully fertile, whereas all of the +1/+1 plants were male‐sterile, and the targeted mutations were inherited in a Mendelian fashion (Table 3). Additionally, the partially male‐sterile GL353‐119 line produced a T_1_ seed through selfing. The T_1_ plant grown from this seed had the parental genotype (+1/−3), and it too was partially male‐sterile.

**Table 2:** Summary of genotypes and phenotypes of DsRed/*Cas9*‐negative T_2_ progeny from line T1‐1

Genotype	Number of lines	Male‐sterile
+1/WT	16 (61.5%)	0 (0%)
+1/+1	4 (15.4%)	4 (100%)
WT/WT	6 (23.1%)	0 (0%)
Chi‐squared	1.69*	

* Segregation conforms to a Mendelian 1:2:1 ratio based on the chi‐squared test (*P *=* *0.43).

**Table 3:** Summary of genotypes and phenotypes of T_3_ progeny from line T2‐14

Genotype	Number of lines	Male‐sterile
+1/WT	22 (44%)	0 (0%)
+1/+1	11 (22%)	11 (100%)
WT/WT	17 (34%)	0 (0%)
Chi‐squared	2.16*	

* Segregation conforms to a Mendelian 1:2:1 ratio based on the chi‐squared test (*P *=* *0.34).

## Discussion

The CRISPR/Cas9 system is a powerful tool for studying gene function and generating genetic diversity in crops. In this study, our aim was to demonstrate the utility of the CRISPR/Cas9 system for the rapid generation of nuclear male sterility in hexaploid wheat. We hypothesized that knockout of *Ms1* through the introduction of targeted biallelic frameshift mutations would result in male sterility, a trait that has high agronomic value for hybrid seed production.

Five per cent (2 of 40) of T_0_ plants carrying gRNA LTPG1‐2 were biallelic mutants, while the majority of the plants (26 of 40) were chimeras in which only a small proportion of cells were edited. Similar editing efficiencies have been reported for gRNAs targeting other wheat genes such as *TaMLO‐A1* (Wang *et al*., 2014) and *TaGW2* (Zhang *et al*., 2016). We observed substantial variation in editing efficiencies between different gRNAs; of the three gRNAs tested, only one (LTPG1‐2) had sufficient activity to generate heritable targeted mutations in *Ms1*. *In silico* prediction of gRNA activity was carried out using the sgRNA Designer (Doench *et al*., 2016) and WU‐CRISPR (Wong *et al*., 2015) tools. These tools use different algorithms to calculate an activity score for each gRNA, where higher scores indicate higher predicted gRNA activity. WU‐CRISPR has a score range of 0–100, but only gRNAs with scores ranging from 50 to 100 are displayed in the database as they are considered ‘good’ candidates (Xiaowei Wang, personal communication). LTPG1‐2 was predicted by WU‐CRISPR to be a good candidate (activity score = 70), whereas LTPG1‐1 and LTPG1‐4 (activity scores < 50) were not. Our experimental results were in agreement with these predictions. sgRNA Designer has a score range of 0–1, and there is no threshold for what is considered a ‘good’ candidate. LTPG1‐1 (activity score = 0.4) was predicted by sgRNA Designer to be the most active of the three gRNAs, but our experimental results were not in agreement with this prediction. The lack of correlation between prediction and experiment in this case is not surprising, as we tested only three gRNAs. Given the variability in gRNA efficacy, and considering the time‐consuming and laborious nature of wheat transformation and tissue culture, it is recommended that gRNAs be validated through transient expression in wheat protoplasts prior to commencing plant transformation (Shan *et al*., 2014).

We did not observe any off‐target editing of *Ms‐A1* or *Ms‐D1* in T_0_ lines. In the case of LTPG1‐2, the presence of multiple mismatches (including at least one mismatch in the critical PAM‐proximal ‘seed’ region of the protospacer) was sufficient to abolish gRNA activity, as expected (Hsu *et al*., 2013; O'Geen *et al*., 2015; Sternberg *et al*., 2014; Wu *et al*., 2014). Thus, LTPG1‐2 shows high specificity for *Ms1* on chromosome 4BS. In the case of LTPG1‐1 and LTPG1‐4, on‐target activity was very low or undetectable, and therefore, the lack of off‐target activity was also expected.

We used three different methods for detecting targeted mutations in transgenic T_0_ plants: capillary separation of fluorescently labelled amplicons, TIDE analysis of Sanger sequence traces and CRISPResso analysis of NGS reads. Only NGS had sufficient sensitivity to detect the low‐frequency targeted mutations in the chimeras, but the three methods produced very similar results for the two biallelic mutants. Therefore, capillary separation of fluorescently labelled homoeolog‐specific amplicons and TIDE analysis of homoeolog‐specific Sanger sequence traces are rapid, reliable and cost‐effective options for initial screening of T_0_ plants for targeted mutations that are likely to be heritable.

As expected, biallelic knockout of *Ms1* in the T_0_ line FL353‐19 (+1/+1) resulted in complete male sterility. By contrast, incomplete knockout of *Ms1* in the biallelic mutant T_0_ line GL353‐119 (+1/−3) resulted in partial male sterility. The targeted mutations carried by GL353‐119 were inherited in a Mendelian fashion, and completely male‐sterile (+1/+1) mutants were recovered in the T_2_ and T_3_ generations, along with fully fertile +1/WT and WT/WT plants. These results are consistent with previous reports of male sterility in EMS‐derived *Ms1* knockout mutants (Tucker *et al*., 2017; Wang *et al*., 2017). Furthermore, we obtained four T_2_ knockout mutants that were apparently non‐transgenic based on fluorescence microscopy and PCR assays. The identification of these mutants was streamlined by employing a fluorescence‐based seed sorting strategy, similar to that which has been developed for Arabidopsis (Gao *et al*., 2016). Interestingly, the partial male‐sterile phenotype, observed in GL353‐119, was inherited by a +1/−3 T_1_ plant. This suggests that the Leu7 residue (deleted in the −3 allele) is required for proper functioning of the signal peptide in Ms1.

Genome editing has been successfully applied for the generation of male‐sterile rice and sorghum lines (Chang *et al*., 2016; Cigan *et al*., 2017; Li *et al*., 2016; Zhou *et al*., 2016). Initial attempts to use (meganuclease‐based) genome editing for the generation of male‐sterile wheat lines (Cigan *et al*., 2017) were met with limited success, as editing of only one of the three *Ms26* homoeologs (A, B or D‐genome) did not confer male sterility due to functional redundancy of the gene. However, conventional crossing of single‐genome biallelic *Ms26* mutants was successfully carried out to produce triple biallelic *Ms26* mutants exhibiting male sterility (Singh *et al*., 2017). More recently, the CRISPR/Cas9 system was used to edit the wheat male fertility gene *Ms45*, and by selfing a triple monoallelic mutant T_1_ plant, male‐sterile triple biallelic mutant T_2_ plants were recovered (Singh *et al*., 2018). Thus, gene functional redundancy can slow the process of recovering edited wheat lines with the desired phenotype, as the targeted mutations often need to be combined and/or made homozygous via conventional breeding.

*Ms1* is a single copy gene located on chromosome 4BS (Tucker *et al*., 2017), and the homoeologs *Ms‐A1* and *Ms‐D1* are epigenetically silenced (Wang *et al*., 2017). This lack of functional redundancy among homoeologs makes *Ms1* a particularly attractive target for genome editing. Indeed, our results demonstrate that the CRISPR/Cas9 system can be used to generate *Ms1* knockout wheat lines that exhibit male sterility in the first generation. We also report here the sequence for LTPG1‐2, an active gRNA that specifically targets *Ms1* in cv. Fielder and cv. Gladius. Further studies will be needed to determine the efficacy of LTPG1‐2 in other wheat cultivars, including elite germplasm from different breeding pools. If LTPG1‐2 is found to be ineffective in a particular target cultivar, for example due to gene functional redundancy, then a different gRNA or multiple gRNAs may be needed. Furthermore, as we only tested three gRNAs, it is likely that screening of additional gRNAs would lead to the identification of a gRNA(s) that exhibits even higher activity than that observed for LTPG1‐2.

Our study provides a methodological foundation and molecular tools for the rapid development of nuclear male‐sterile wheat lines. This represents a significant step toward the establishment of a commercially viable hybrid wheat platform. Thus, we anticipate that the adoption of genome editing technologies for precision wheat breeding, together with a better understanding of wheat floral architecture and the flower opening process (Okada *et al*., 2018), will ultimately lead to increased yield gains through capturing heterosis.

## Experimental procedures

### gRNA design

Partial *Ms1*,* Ms‐A1* and *Ms‐D1* sequences derived from *T. aestivum* cultivars Fielder and Gladius were used for gRNA design. The gRNAs were designed to target exon 1 of *Ms1* on chromosome 4BS, in a region immediately downstream of the start codon, based on the presence of the canonical PAM (5′‐NGG‐3′). Guide sequences were 20 nucleotides in length. To ensure efficient transcription from the TaU6 promoter, all gRNAs had a G nucleotide at position +20 (PAM‐distal end) of the guide sequence (Sander and Joung, 2014).

### Vector design and construction

All vectors were designed using Vector NTI software. The gRNA expression cassette (Shan *et al*., 2013) consisting of the TaU6 promoter and a non‐targeting (random guide sequence) gRNA was synthesized (GenScript) and cloned into pUC57, resulting in pUC57‐gRNA. Annealed oligos containing the *Ms1*‐targeting guide sequence (Table S2) were cloned into pUC57‐gRNA by simultaneous digestion/ligation with *BbsI* and T4 DNA ligase. Positive clones were identified by diagnostic restriction digest and validated by Sanger sequencing (Australian Genome Research Facility).

The rice codon‐optimized *Sp*Cas9 gene with N‐ and C‐terminal nuclear localization signals (Shan *et al*., 2013) was synthesized (GenScript) and inserted into PHP62407M as an *NcoI*–*AscI* fragment between the maize *Ubi1* promoter and the *Sorghum bicolor* actin terminator, resulting in the entry vector pCas9‐NB.

The *Agrobacterium* T‐DNA binary vector pMDC123 (Curtis and Grossniklaus, 2003) was modified by replacing the original selection cassette with an intron‐containing *bar* gene regulated by the maize *Ubi1* promoter and the wheat *rbcS* Class II terminator. In addition, an aleurone‐specific fluorescent reporter (DsRed2) cassette (Wu *et al*., 2016) was inserted between the Gateway cassette and the right border, resulting in the destination vector pMDC‐Bar‐DsRed. The Cas9 expression cassette from pCas9‐NB was Gateway cloned into pMDC‐Bar‐DsRed to produce the intermediate vector pNB1.

Finally, the gRNA expression cassettes from three different pUC57‐gRNA vectors pre‐loaded with *Ms1*‐targeting guide sequences (LTPG1‐1, LTPG1‐2 and LTPG1‐4, respectively) were individually cloned into pNB1 as *AsiSI*‐*PmeI* fragments between the DsRed2 expression cassette and the right border. The resulting T‐DNA binary vectors (pNB‐LTPG1‐1, pNB‐LTPG1‐2 and pNB‐LTPG1‐4) were used for *Agrobacterium*‐mediated transformation.

### *Agrobacterium*‐mediated transformation

Transformation of cv. Fielder and cv. Gladius was carried out as described (Ishida *et al*., 2015), with minor modifications. Briefly, immature embryos were isolated from spikes harvested at 14 days post‐anthesis. Isolated embryos were transferred to WLS‐liq solution, centrifuged at 16 000 ***g*** for 10 min, incubated in WLS‐inf solution containing *Agrobacterium* (strain AGL1) for 5 min and then transferred to WLS‐AS media for 2 days of co‐cultivation. After co‐cultivation, embryo axes were removed, and then, scutella were transferred to WLS‐Res media for 5 days of resting culture. After the resting culture, scutella were transferred to WLS‐P5 callus induction media (selection with 5 mg/L phosphinothricin) for 2 weeks, followed by WLS‐P10 callus induction media (selection with 10 mg/L phosphinothricin) for 3 weeks. Calli were then transferred to LSZ‐P5 regeneration media (selection with 5 mg/L phosphinothricin) for 2 weeks under a cycle of 12 h dark/12 h light (~70 μmol/m^2^/s). Regenerants were transferred to LSF‐P5 rooting media (selection with 10 mg/L phosphinothricin) for 2 weeks, before being transferred to potted soil in the greenhouse. Timentin was substituted for cefotaxime in all tissue culture media.

### Detection of targeted mutations by capillary separation of fluorescently labelled amplicons

Genomic DNA was extracted from the second leaves of transgenic T_0_ plants at the vegetative stage, using a freeze‐dried method (Kovalchuk, 2014). The target site was amplified by PCR using Phusion High‐Fidelity DNA Polymerase (New England BioLabs), Phusion GC Buffer, 5% DMSO, 1 m betaine and a pair of *Ms1*‐specific 6‐FAM‐labelled primers Table (S2). To generate wild‐type amplicons for spike‐in (size reference), the same primer pair labelled with HEX was used. Touchdown PCR cycling conditions were as follows: initial denaturation at 98 °C for 3 min, denaturation at 98 °C for 15 s, annealing at 70–65 °C for 20 s, extension at 72 °C for 15 s and final extension at 72 °C for 5 min. The starting annealing temperature was decreased by 0.5 °C each cycle for 10 cycles, followed by 25 cycles at the final annealing temperature. A sample of the PCR product was run on an agarose gel to confirm the presence of a single band of the expected size (432 bp). The fluorescently labelled amplicons were diluted and subjected to capillary electrophoresis (Australian Genome Research Facility) on an AB3730 DNA Analyzer (Applied Biosystems, Foster City, CA). The results were analysed using PeakScanner Software 2 (Applied Biosystems). In PeakScanner, the peak for the HEX‐labelled wild‐type size reference was adjusted to a fluorescence intensity of approximately 3000, to improve visual clarity.

### Detection of targeted mutations by Sanger sequencing and TIDE

Genomic DNA was extracted from transgenic T_0_ plants as described above. The target site was amplified by PCR using Phusion High‐Fidelity DNA Polymerase, Phusion GC Buffer, 5% DMSO, 1 m betaine and a pair of *Ms1*‐specific primers Table (S2). Touchdown PCR cycling conditions were as follows: initial denaturation at 95 °C for 8 min, denaturation at 94 °C for 10 s, annealing at 62–57 °C for 30 s, extension at 72 °C for 30 s and final extension at 72 °C for 5 min. The starting annealing temperature was decreased by 0.5 °C each cycle for 10 cycles, followed by 30 cycles at the final annealing temperature. A sample of the PCR product was run on an agarose gel to confirm the presence of a single band of the expected size (577 bp). The amplicons were then column‐purified and Sanger sequenced (Australian Genome Research Facility) on a 3730xl DNA Analyzer (Applied Biosystems). Bases were called with KB Basecaller v1.4.1.8, and the AB1 files were uploaded to the online TIDE analysis tool (Brinkman *et al*., 2014). In TIDE, the indel size range was set at 10, and the other settings were adjusted based on information provided on the online TIDE analysis tool Troubleshooting webpage. The proportion of edited DNA in the sampled tissue was calculated as the sum of all significant indels (*P *<* *0.001) detected by TIDE.

### Detection of targeted mutations by NGS and CRISPResso analysis

Genomic DNA was extracted from transgenic T_0_ plants as described above. To generate amplicons for NGS, two rounds of PCR were carried out. The PCR mixtures contained Phusion High‐Fidelity DNA Polymerase, Phusion GC Buffer, 5% DMSO and 1 m betaine. In the first round of PCR, the target site was amplified using conserved primers flanked by 5′ universal tail sequences Table (S2). PCR cycling conditions were as follows: initial denaturation at 98 °C for 3 min, followed by 30 cycles of 98 °C for 20 s, 72 °C for 20 s, with a final extension at 72 °C for 2 min. A sample of the PCR product was run on an agarose gel to confirm the presence of bands of the expected sizes (300 bp, 313 bp, 327 bp). In the second round of PCR, barcodes and adapters were added using Illumina Nextera XT primers that anneal to the tail sequences of the primers used in the first round of PCR. Cycling conditions in the second round of PCR were as follows: initial denaturation at 98 °C for 3 min, followed by six cycles of 98 °C for 30 s, 55 °C for 30 s, 72 °C for 30 s, with a final extension of 72 °C for 5 min. The barcoded PCR products were purified using Agencourt AMPure XP beads (Beckman Coulter), quantified by qPCR, pooled in equimolar amounts, spiked with 10% PhiX Control v3 and then sequenced (Australian Genome Research Facility) on the Illumina MiSeq platform using the MiSeq Reagent Kit v3 300 cycle. The raw reads from each sample were filtered using *Ms1*,* Ms‐A1* and *Ms‐D1*‐specific tag sequences Table (S2) and assigned to their respective homoeologs. FASTQ files containing the homoeolog‐specific reads were used as input for the CRISPResso analyses (Pinello *et al*., 2016). In CRISPResso, the following parameters were used: –w 20 –hide_mutations_outside_window_NHEJ –save_also_png –trim_sequences ‐q 30 –exclude_bp_from_left 5 –exclude_bp_from_right 5 –ignore_substitutions. Allele frequencies in Figure 3 were calculated by summing the values in the %Reads column of the CRISPResso allele frequency table, after filtering out aligned sequences that did not contain the partial allele sequence shown. Editing frequencies in Table 1 were calculated using data from the CRISPResso pie charts.

### Pollen viability assay

Pollen viability was assessed by Lugol (1% I_3_K solution) staining. Pollen grains were mounted on glass microscope slides and imaged using a Nikon Ni‐E microscope equipped with a DS‐Ri1‐U3 camera (Adelaide Microscopy Waite Facility). Images were captured with NIS‐Elements software.

### DsRed expression assay

Mature seeds were imaged with a Leica MZ FLIII microscope equipped with a Leica DFC450 C digital camera and a DsRed filter set. Images were captured with Leica Application Suite v4.10.0 software.

### Detection of the Cas9 transgene in T_2_ plants

Genomic DNA was extracted from T_2_ plants as described above. The Cas9 transgene was detected by PCR using Phusion High‐Fidelity DNA Polymerase, Phusion HF Buffer and a pair of *Cas9*‐specific primers Table (S2). Touchdown PCR cycling conditions were as follows: initial denaturation at 98 °C for 2 min, denaturation at 98 °C for 15 s, annealing at 72–69 °C for 20 s, extension at 72 °C for 15 s and final extension at 72 °C for 5 min. The starting annealing temperature was decreased by 0.5 °C each cycle for 6 cycles, followed by 24 cycles at the final annealing temperature. A sample of the PCR product was run on an agarose gel to check for the presence/absence of the expected 281 bp band.

### *AluI* assay for genotyping T_2_ and T_3_ plants

Genomic DNA was extracted from T_2_ and T_3_ plants as described above. The target site was amplified by PCR using Phusion High‐Fidelity DNA Polymerase, Phusion GC Buffer, 5% DMSO, 1 m betaine and pair of *Ms1*‐specific primers Table (S2). Touchdown PCR cycling conditions were as follows: initial denaturation at 95 °C for 8 min, denaturation at 94 °C for 10 s, annealing at 70–65 °C for 30 s, extension at 72 °C for 20 s and final extension at 72 °C for 5 min. The starting annealing temperature was decreased by 0.5 °C each cycle for 10 cycles, followed by 27 cycles at the final annealing temperature. A sample of the PCR product was run on an agarose gel to confirm the presence of a single band of the expected size (432 bp). Five μL of unpurified amplicons was digested with 2 units of *AluI* in a 7 μL reaction and then run on a 2% agarose gel.

### Statistics

To test for Mendelian inheritance of edited alleles in the T_2_ and T_3_ generations, Pearson's chi‐squared test was used, as described (Montoliu, 2012).

### Post hoc *in silico* prediction of gRNA activity

gRNA on‐target activity was predicted using the sgRNA Designer (Doench *et al*., 2016) and WU‐CRISPR (Wong *et al*., 2015) tools, according to the developers’ guidelines.

## Conflict of interest

ET, UB and RW have filed a patent on previous published work on *Ms1*. The remaining authors declare no conflict of interest.

## GenBank accession numbers

Partial *Ms1* sequence obtained from cultivar Fielder: MK039721; Partial *Ms‐A1* sequence obtained from cultivar Fielder: MK039722; Partial *Ms‐D1* sequence obtained from cultivar Fielder: MK039723; Partial *Ms1* sequence obtained from cultivar Gladius: MK039724; Partial *Ms‐A1* sequence obtained from cultivar Gladius: MK039725; Partial *Ms‐D1* sequence obtained from cultivar Gladius: MK039726.

## Supporting information


**Table S1** Transgene copy numbers for T_0_ lines.
**Table S2** Primers, oligonucleotides and sequence tags used in this study.
**Figure S1** Pollen viability assay based on detection of starch via iodine‐potassium iodide staining. Wild type cv. Gladius (left) and the partially male‐sterile edited line GL353‐119 (right) are shown. Scale bar = 100 μm.

**Data S1** *Ms1* Sanger sequence trace for cv. Fielder transgenic T_0_ line FL353‐9.

**Data S2** *Ms1* Sanger sequence trace for cv. Fielder transgenic T_0_ line FL353‐14.

**Data S3** *Ms1* Sanger sequence trace for cv. Fielder transgenic T_0_ line FL353‐19.

**Data S4** *Ms1* Sanger sequence trace for wild type cv. Fielder.

**Data S5** *Ms1* Sanger sequence trace for cv. Gladius transgenic T_0_ line GL353‐45.

**Data S6** *Ms1* Sanger sequence trace for cv. Gladius transgenic T_0_ line GL353‐48.

**Data S7** *Ms1* Sanger sequence trace for cv. Gladius transgenic T_0_ line GL353‐61.

**Data S8** *Ms1* Sanger sequence trace for cv. Gladius transgenic T_0_ line GL353‐69.

**Data S9** *Ms1* Sanger sequence trace for cv. Gladius transgenic T_0_ line GL353‐82.

**Data S10** *Ms1* Sanger sequence trace for cv. Gladius transgenic T_0_ line GL353‐119.

**Data S11** *Ms1* Sanger sequence trace for cv. Gladius transgenic T_0_ line GL353‐121.

**Data S12** *Ms1* Sanger sequence trace for cv. Gladius transgenic T_0_ line GL353‐123.

**Data S13** *Ms1* Sanger sequence trace for cv. Gladius transgenic T_0_ line GL353‐124.

**Data S14** *Ms1* Sanger sequence trace for wild type cv. Gladius.
