# Supplementary material for: CRISPR/Cas9‐mediated knockout of Ms1 enables the rapid generation of male‐sterile hexaploid wheat lines for use in hybrid seed production
Source: Plant Biotechnol J. 2019 Apr 11;17(10):1905–13. doi: 10.1111/pbi.13106 (PMC6737020; doi:10.1111/pbi.13106)
Supplement: Supplementary file 1 — Table S1 Transgene copy numbers for T0 lines. Table S2 Primers, oligonucleotides and sequence tags used in this study. Figure S1 Pollen viability assay based on detection of starch via iodine‐potassium iodide staining. Wild type cv. Gladius (left) and the partially male‐sterile edited line GL353‐119 (right) are shown. Scale bar = 100 μm. [file PBI-17-1905-s015.docx]

**Table S1** Transgene copy numbers for T_0_ lines

| **gRNA** | **Plant ID** | **Copy Number** |
| --- | --- | --- |
| LTPG1-1 (n = 18) | FL352_T0_1 | 1 |
|  | FL352_T0_2 | 5 |
|  | FL352_T0_4 | 1 |
|  | FL352_T0_5 | 5 |
|  | FL352_T0_6 | 1 |
|  | FL352_T0_7 | 5 |
|  | FL352_T0_8 | 1 |
|  | FL352_T0_9 | 5 |
|  | FL352_T0_10 | 1 |
|  | FL352_T0_11 | 5 |
|  | FL352_T0_12 | 1 |
|  | FL352_T0_13 | 5 |
|  | FL352_T0_14 | 1 |
|  | FL352_T0_15 | 5 |
|  | FL352_T0_18 | 1 |
|  | FL352_T0_20 | 5 |
|  | FL352_T0_21 | 1 |
|  | FL352_T0_22a | 5 |
| LTPG1-2 (n = 40) | GL353_T0_45 | 1 |
|  | GL353_T0_46 | 1 |
|  | GL353_T0_47 | 3 |
|  | GL353_T0_48 | 1 |
|  | GL353_T0_49 | 2 |
|  | GL353_T0_54 | 4 |
|  | GL353_T0_59 | 13 |
|  | GL353_T0_61 | 14 |
|  | GL353_T0_63 | 2 |
|  | GL353_T0_64 | 1 |
|  | GL353_T0_66 | 4 |
|  | GL353_T0_67 | 16 |
|  | GL353_T0_68 | 2 |
|  | GL353_T0_69 | 2 |
|  | GL353_T0_70 | 1 |
|  | GL353_T0_72 | 1 |
|  | GL353_T0_82 | 2 |
|  | GL353_T0_106 | 1 |
|  | GL353_T0_117 | 3 |
|  | GL353_T0_118 | 1 |
|  | GL353_T0_119 | 12 |
|  | GL353_T0_121 | 12 |
|  | GL353_T0_122 | 5 |
|  | GL353_T0_123 | 6 |
|  | GL353_T0_124 | 3 |
|  | GL353_T0_125 | 2 |
|  | GL353_T0_126 | 2 |
|  | GL353_T0_127 | 2 |
|  | GL353_T0_128 | 11 |
|  | GL353_T0_129 | 1 |
|  | FL353_T0_3 | 1 |
|  | FL353_T0_5 | 1 |
|  | FL353_T0_6 | 1 |
|  | FL353_T0_7 | 2 |
|  | FL353_T0_8 | 2 |
|  | FL353_T0_9 | 1 |
|  | FL353_T0_14 | 3 |
|  | FL353_T0_17 | 1 |
|  | FL353_T0_19 | 7 |
|  | FL353_T0_30 | 2 |
| LTPG1-4 (n = 16) | GL360_T0_24 | 18 |
|  | GL360_T0_42 | 12 |
|  | GL360_T0_64 | 10 |
|  | GL360_T0_81 | 8 |
|  | GL360_T0_82 | 6 |
|  | GL360_T0_83 | 1 |
|  | FL360_T0_5 | 1 |
|  | FL360_T0_6 | 2 |
|  | FL360_T0_8 | 4 |
|  | FL360_T0_19 | 2 |
|  | FL360_T0_24 | 1 |
|  | FL360_T0_27 | 8 |
|  | FL360_T0_48 | 2 |
|  | FL360_T0_67 | 1 |
|  | FL360_T0_84 | 3 |
|  | FL360_T0_89 | 1 |

**Table S2** Primers, oligonucleotides and sequence tags used in this study

| Oligo name | Sequence 5'-3' (non-binding in red) | Use |
| --- | --- | --- |
| TaLTPG1-1_F | CTTGAGATCCCGCGGGCTGCTGC | Sense oligo for gRNA LTPG1-1 |
| TaLTPG1-1_R | AAACGCAGCAGCCCGCGGGATCT | Antisense oligo for gRNA LTPG1-1 |
| TaLTPG1-2_F | CTTGCGCCACCAGCAGCAGCCCG | Sense oligo for gRNA LTPG1-2 |
| TaLTPG1-2_R | AAACCGGGCTGCTGCTGGTGGCG | Antisense oligo for gRNA LTPG1-2 |
| TaLTPG1-4_F | CTTGCTGCTGGCGGCGCTGCTGC | Sense oligo for gRNA LTPG1-4 |
| TaLTPG1-4_R | AAACGCAGCAGCGCCGCCAGCAG | Antisense oligo for gRNA LTPG1-4 |
| ET0495_F5 | GCATTCCATTCCGCCACCG | Fwd primer for amplification of target sites on 4BS (capillary separation and AluI assay) |
| ET0514_R_FAM | [FAM6]GGGAGGAGGCGGACAACGTA | Rev primer (FAM-labelled) for amplification of target sites on 4BS (capillary separation) |
| ET0514_R_HEX | [HEX]GGGAGGAGGCGGACAACGTA | Rev primer (HEX-labelled) for amplification of target sites on 4BS (capillary separation) |
| ET0495_F6 | CCCCTCCGGCATTCCATTC | Fwd primer for amplification of target site on 4BS (Sanger sequencing) |
| ET0493_R7 | AGATCCGGTAGTACAGCGAGGAAATTAACAG | Rev primer for amplification of target site on 4BS (Sanger sequencing) |
| ET495_R4 | CTACTAGGTAAGCAGGAAAGCGAGCA | Sanger sequencing primer (Gladius lines) |
| 007.0091.7.C1 | GGGAGGAGGCGGACAACGTA | Sanger sequencing primer (Fielder lines), Rev primer for amplification of target site on 4BS (AluI assay). |
| TaLTPG_NGS_F1_P5_3044 | TCGTCGGCAGCGTCAGATGTGTATAAG AGACAGCTCCCCCTCCGGCATTCCA | Primer for first round of amplification for NGS. Used with TaLTPG_NGS_R3_P7_3303 |
| TaLTPG_NGS_R3_P7_3303 | GTCTCGTGGGCTCGGAGATGTGTATAAGAGACAGGGCATGTCGGGCGCGCAGAAG | Primer for first round of amplification for NGS. Used with TaLTPG_NGS_F1_P5_3044 |
| TaLTPG_NGS_F3_P5_3033 | TCGTCGGCAGCGTCAGATGTGTATAAG AGACAGGGCATGTCGGGCGCGCAGAAG | Primer for first round of amplification for NGS. Used with TaLTPG_NGS_R2_P7_3044 |
| TaLTPG_NGS_R2_P7_3044 | GTCTCGTGGGCTCGGAGATGTGTATAAGAGACAGCTCCCCCTCCGGCATTCCA | Primer for first round of amplification for NGS. Used with TaLTPG_NGS_F3_P5_3033 |
| TaLTPG_B genome_1^st^ tag | TTCCGCCACCGCA | Identification of NGS reads derived from 4BS |
| TaLTPG_B genome_2^nd^ tag | GGCGGCGGCGCAG | Identification of NGS reads derived from 4BS |
| TaLTPG_D genome_1^st^ tag | CCCCAACACCGCA | Identification of NGS reads derived from 4DS |
| TaLTPG_D genome_2^nd^ tag | GTTCGGGCAGCAG | Identification of NGS reads derived from 4DS |
| TaLTPG_A genome_1^st^ tag | TTTCCGTCCCACC | Identification of NGS reads derived from 4AL |
| TaLTPG_A genome_2^st^ tag | CTTCGGGCAGCAG | Identification of NGS reads derived from 4AL |
| Cas-F1 | GGGACAAGCCTATCAGAGAGC | Fwd primer for detection of Cas9 transgene |
| Cas-R1 | GCGCGCCTAGTTAGTTAGTCAC | Rev primer for detection of Cas9 transgene |


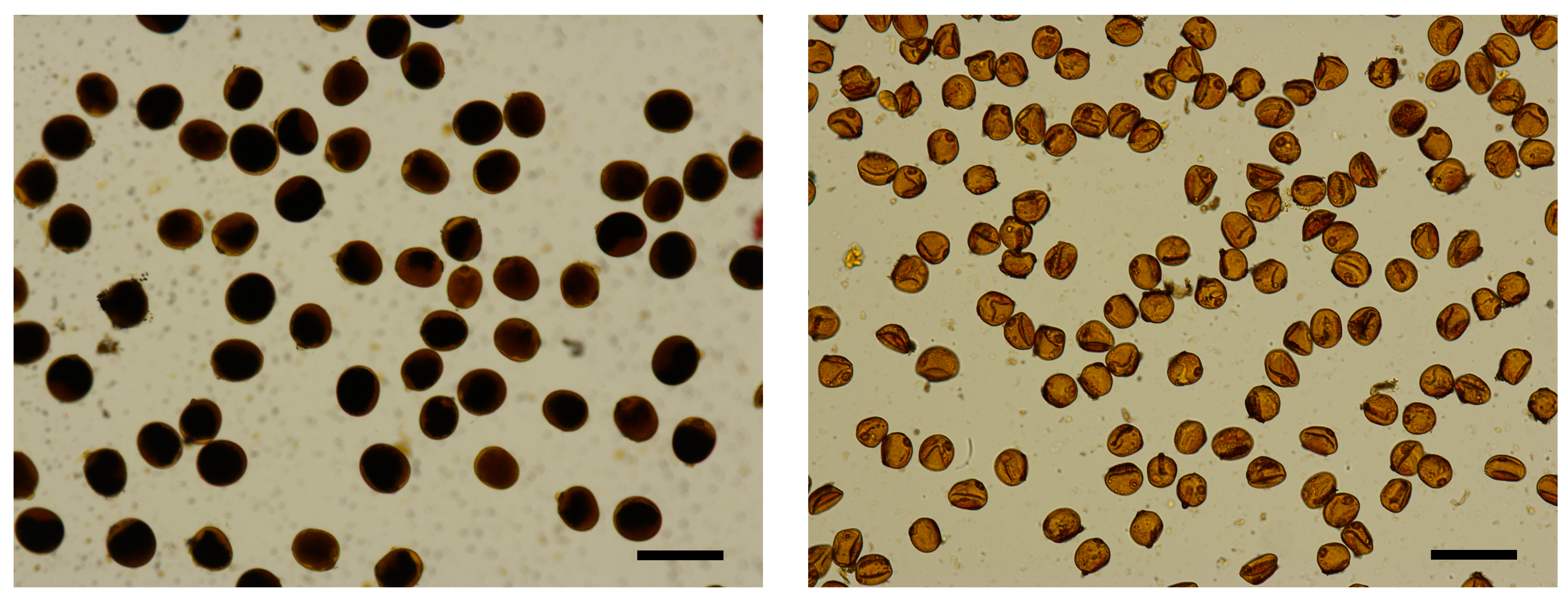


**Figure S1** Pollen viability assay based on detection of starch via iodine-potassium iodide staining. Wild type cv. Gladius (left) and the partially male-sterile edited line GL353-119 (right) are shown. Scale bar = 100 µm.
